# Omics research in Parkinson’s disease: evolution, integrated analysis, pathogenic mechanisms, biomarkers, and therapeutic targets

**DOI:** 10.3389/fnmol.2026.1821875

**Published:** 2026-07-16

**Authors:** Ruizhe Cao, Xue Bai, Huan Zhong

**Affiliations:** 1 School of Nursing, Tongji Medical College, Huazhong University of Science and Technology, Wuhan, China; 2 Department of Biochemistry and Molecular Biology, University of British Columbia, Vancouver, BC, Canada

**Keywords:** biomarkers, integrative multi-omics analysis, interventional targets, Parkinson’s disease, pathogenic mechanisms

## Abstract

Parkinson’s Disease (PD) is a common neurodegenerative disorder that has been widely investigated using omics approaches over 2020–2025. This review focuses on the transition from single-omics to integrative multi-omics analysis, which helps overcome the constraints of individual omics strategies and provides a more systematic understanding of PD pathogenesis. Key topics include the elucidation of core mechanisms (mitochondrial dysfunction, lipid metabolic disturbance, *α*-synucleinopathy, and gut–brain axis impairment), the development of biomarkers (shifting from invasive cerebrospinal fluid samples to less invasive plasma/urine panels with improved performance), and the identification of potential interventional targets (signaling pathways, key molecules, and gut microbiota). Technical advances include refined single-omics detection, AI-assisted multi-omics integration, and the emergence of multi-center large cohorts. Despite notable progress, major challenges remain, including limited sample diversity, insufficient technical standardization, and gaps between basic research and clinical translation. Future directions should prioritize the establishment of multi-center longitudinal biobanks, high-sensitivity detection methods, and clinical translation through precision subtyping and targeted intervention. Integrative multi-omics has significantly advanced PD research from fragmented studies to a more systematic framework, providing a foundation for early diagnosis and precision therapy. Further efforts are needed to address current limitations and translate findings into clinical benefit for PD patients.

## 1 Introduction

PD is a neurodegenerative disorder primarily caused by the degeneration of dopamine-producing neurons in the substantia nigra (SN) of the brain (Shokrpour et al., 2025). This results in the emergence of motor and non-motor symptoms. As dopamine acts as a neurotransmitter that transduces signals between the SN and the striatum of the brain, and the nigrostriatal pathway modulates motor activity and enables voluntary movement, the motor symptoms associated with dopamine deficiency include tremor, bradykinesia, postural instability, and gait impairment, among others (Shokrpour et al., 2025). Non-motor symptoms secondary to dopamine deficiency include speech impairment, depression, fatigue, and anxiety, among others (Dhanalakshmi et al., 2024). The pathological hallmarks of PD encompass the presence of Lewy bodies and Lewy neurites, as well as the loss of dopaminergic neurons in the SN; since misfolded *α*-Synuclein aggregates constitute the major component of Lewy bodies is thus classified as α-Synucleinopathy (Tolosa et al., 2021). PD is classified into sporadic and familial forms based on genetics and pathogenesis, with some genetic features and clinical symptoms shared between the two categories (Karimi-Moghadam et al., 2018). Sporadic Parkinson’s disease (sPD) represents the most prevalent clinical subtype with no familial inheritance history. Monogenic Parkinson’s disease can also occur in sporadic cases. Its pathogenesis results from the combined effects of genetic susceptibility, environmental exposure and aging, and familial aggregation may be observed occasionally (Dulski et al., 2022); sPD can be categorized into subtypes based on onset age, clinical manifestations and disease progression, with symptoms varying among individual patients (Qian and Huang, 2019). Familial Parkinson’s disease presents a clear familial hereditary lineage and is induced by specific pathogenic gene mutations, with diverse genetic transmission patterns and prominent familial clustering of morbidity. Multiple subtypes can be classified according to disease-causing genes, among which subtypes related to PARK1, PARK2, PARK6 and PARK8 are prevalent. Patients with this disease generally have an earlier onset age. Their disease progression rate, symptom severity and response to pharmacological treatment differ substantially from those with sporadic Parkinson’s disease, and clinical manifestations are closely associated with pathogenic genes (Jia et al., 2022).

Based on existing research, age is an important risk factor for PD and the disease is also associated with gender, with males having a higher susceptibility to PD than females (Tolosa et al., 2021). However, there are no specific diagnostic tests capable of accurately identifying the disease nor therapeutic interventions that can achieve a permanent cure for it (Dhanalakshmi et al., 2024); thus, the accurate diagnosis of PD and the advancement of therapeutic research on the disease have become the key focus of addressing this issue.

To address the aforementioned issues, omics analysis has emerged as one of the key approaches for investigating PD, such as genomics, transcriptomics, proteomics, metabolomics, epigenomics, and microbiomics analyses. However, single-omics analysis is unable to systematically and comprehensively elucidate the pathogenic mechanisms of PD: Genomics identifies PD susceptibility loci by detecting variations in DNA sequences (Pan et al., 2023), yet it fails to reflect the dynamic pathological processes of the disease. Transcriptomics assesses the transcriptional activity of PD-associated genes by detecting RNA expression levels (Salemi et al., 2022), yet its results are susceptible to interference from sample heterogeneity. Proteomics directly detects proteins in biological samples (Hällqvist et al., 2024), yet it cannot independently elucidate the underlying causes of protein alterations. Metabolomics identifies small-molecule metabolites (e.g., amino acids, lipids, neurotransmitters, among others) (Plewa et al., 2021), yet it is unable to determine the specific disease etiologies responsible for fluctuations in metabolite levels—such fluctuations can also be triggered by other neurodegenerative disorders (Kurano et al., 2024). Epigenomics (e.g., DNA methylation, histone modification) investigates the regulatory mechanisms that modulate gene expression without altering the DNA sequence (Klokkaris and Migdalska-Richards, 2024), yet it cannot verify whether ‘modulating methylation can reverse PD pathological processes’ via single-omics analysis. Microbiomics profiles the composition of the gut microbiota to identify PD-associated gut microbial taxa (Mehanna et al., 2023), yet it cannot elucidate, using microbiomics alone, whether PD-associated microbial taxa induce neuroinflammation by generating microbial metabolites (e.g., short-chain fatty acids (Bedarf et al., 2025), lipopolysaccharides (Brown et al., 2023)) that enter the bloodstream and cross the blood–brain barrier (Gao A. et al., 2025), or trigger the development of PD through other alternative pathways.

Integrative multi-omics analysis holds profound significance for the investigation, diagnosis and treatment of PD. Its core value resides in overcoming the limitations of single molecular levels: by integrating multi-dimensional information encompassing genetic, transcriptional, proteomic, metabolic, epigenetic regulatory and gut microbial profiles, it enables a more comprehensive elucidation of the complex pathological mechanisms and clinical characteristics of PD. For instance, integrative analysis of proteomics and metabolomics has identified the ‘IL1B-nitric oxide (NO)-NGF’ axis as the core regulatory node underlying PD-associated pathological processes in CSF (Kim et al., 2024). Integrative analysis of proteomics and lipidomics, incorporating the Mendelian randomization (MR) approach, has unveiled the causal associations between lipids, inflammatory proteins and PD as well as their corresponding mediating pathways (Qin et al., 2024). Targeted metabolomic profiling (including lipidome coverage) has identified the dysregulation of core lipid metabolic pathways in the plasma of PD patients, with the activation of sphingolipid metabolism emerging as a key pathological feature (Gątarek and Kałużna-Czaplińska, 2024). Integrative analysis of proteomics and transcriptomics (RNA-seq) has unveiled the asynchrony between mRNA and protein levels in PD pathological processes and shared diagnostic gene panel point to the core pathological crossroads of PD (Dumitriu et al., 2015). Integrative analysis of metabolomics and transcriptomics has unveiled the interorgan metabolic-gene associations between the liver and brain, alongside the key metabolite-gene pairs and shared regulatory pathways implicated in these cross-organ regulatory networks (Hu et al., 2023). This series of multi-omics case studies demonstrates that integrative multi-omics analysis has afforded PD research a research perspective of ‘from fragmentation to systematic integration’.

To clarify the terminology in this review, we hereafter distinguish two categories of omics integration: (1) simple two-omics juxtaposition that only compares differential profiles from two omics layers without joint computational modeling or network integration; (2) rigorous integrative multi-omics that adopts mathematical modeling, network analysis, Mendelian randomization or machine learning to realize systematic data fusion. Only the latter is defined as standard multi-omics integration in this review.

## 2 Methods

**Figure 1:**
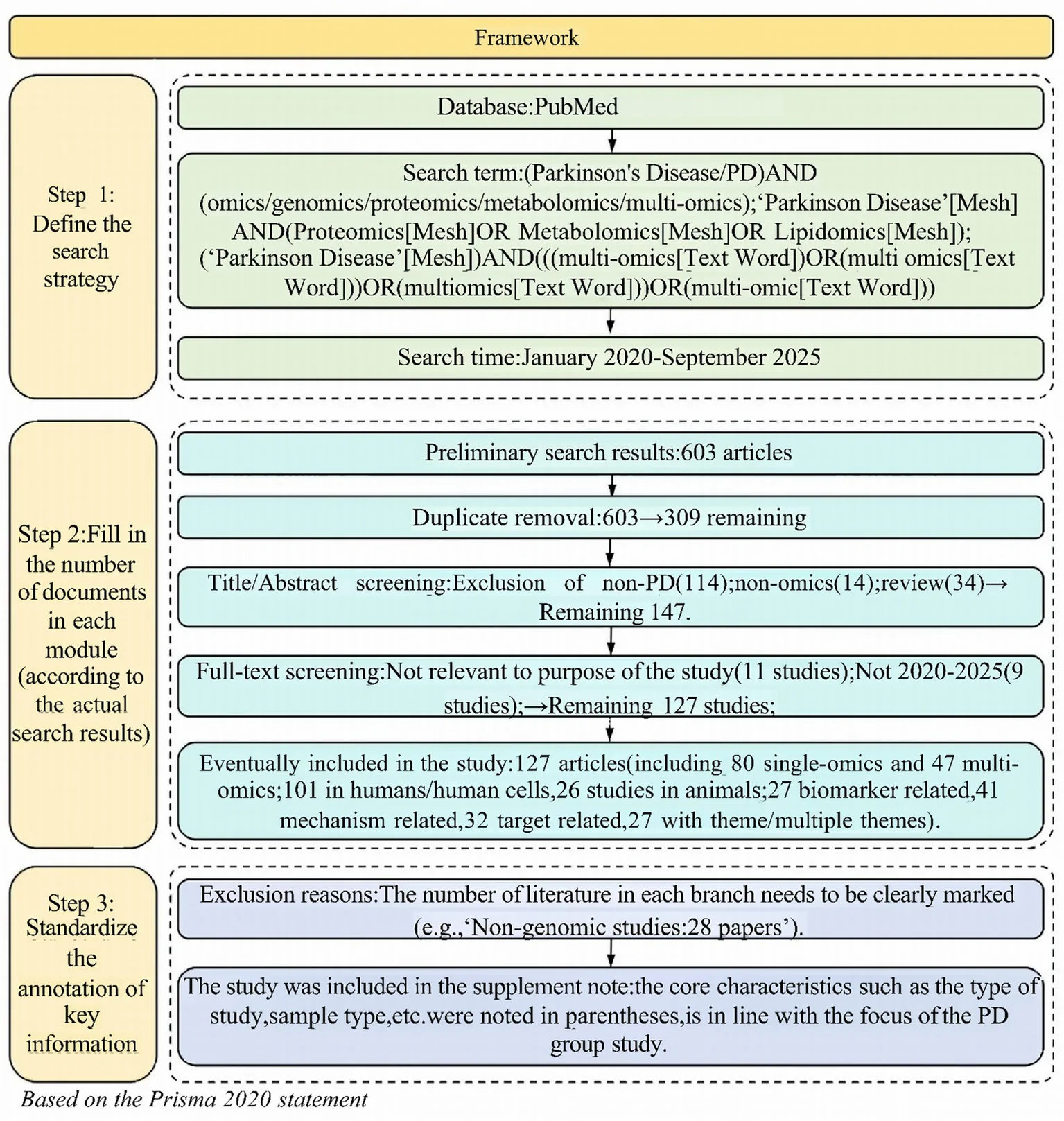
Study flow diagram of literature screening and selection for Parkinson’s disease (PD) omics-related studies. The systematic literature search was conducted across the PubMed databases, covering the period from January 2020 to September 2025 (Supplementary Table S1). The search strategy combined PD-focused terms (e.g., ‘Parkinson’s Disease’ [MeSH]) with omics-related keywords (e.g., ‘multi-omics’, ‘proteomics’ [MeSH]) to ensure comprehensive retrieval. After duplicate removal (reducing 603 initial records to 309), title/abstract screening excluded non-PD studies (*n* = 114), non-omics research (*n* = 14), and review articles (*n* = 34), leaving 147 records for full-text evaluation. Full-text screening further excluded studies irrelevant to the research aim (*n* = 11) and those outside the 2020–2025 timeframe (*n* = 9), resulting in the final inclusion of 127 articles. These were categorized by omics type (80 single-omics, 47 multi-omics), experimental model (101 human/human cell studies, 26 animal studies), and research theme (27 biomarker-focused, 41 mechanism-focused, 32 target-focused, and 27 with multiple overlapping themes). The screening process adheres to the PRISMA 2020 statement, with standardized annotation of exclusion reasons and supplementary notes on core study characteristics (e.g., study design, sample source) for all included records.

This study was conducted in accordance with the PRISMA 2020 Statement. We retrieved relevant literature from PubMed, with the publication period limited to January 2020 to September 2025. Search strategies combined keywords and Medical Subject Headings (MeSH) related to PD and omics. After removing duplicates, we performed title/abstract screening and subsequent full-text evaluation. A total of 127 studies were finally included, consisting of 80 single omics studies and 47 multi-omics studies. During literature screening, we standardized data extraction and recorded reasons for exclusion as well as core characteristics of each included study (e.g., sample type) (Figure 1).

### 2.1 Search strategies

1. *Free terms*: (Parkinson’s Disease OR PD) AND (omics OR genomics OR proteomics OR metabolomics OR multi-omics).
2. *MeSH terms*: ‘Parkinson Disease’ [MeSH] AND (Proteomics [MeSH] OR Metabolomics [MeSH] OR Lipidomics [MeSH]).
3. *Combined MeSH and text words*: ‘Parkinson Disease’ [MeSH] AND (multi-omics [TW] OR multi omics [TW] OR multiomics [TW] OR multi-omic [TW]).

The inclusion criteria were defined as follows: (1) original articles published from January 2020 to September 2025; (2) studies indexed in PubMed; (3) research focusing on PD and omics analyses including genomics, proteomics, metabolomics and multi-omics.

Exclusion criteria were as follows: duplicate publications, studies irrelevant to PD, studies without omics-related analyses, review articles, studies inconsistent with the research objectives, preprints, and publications outside the predefined time frame.

Quality assessment of the included studies was implemented by two independent reviewers. The assessment items covered study design, sample selection, data integrity and result reliability. Any disagreements between the two reviewers were resolved through discussion or adjudication by a third investigator. Notably, journal impact factor was not used as a screening criterion in this study.

## 3 Common core objectives of PD omics research (2020–2025)

**Figure 2:**
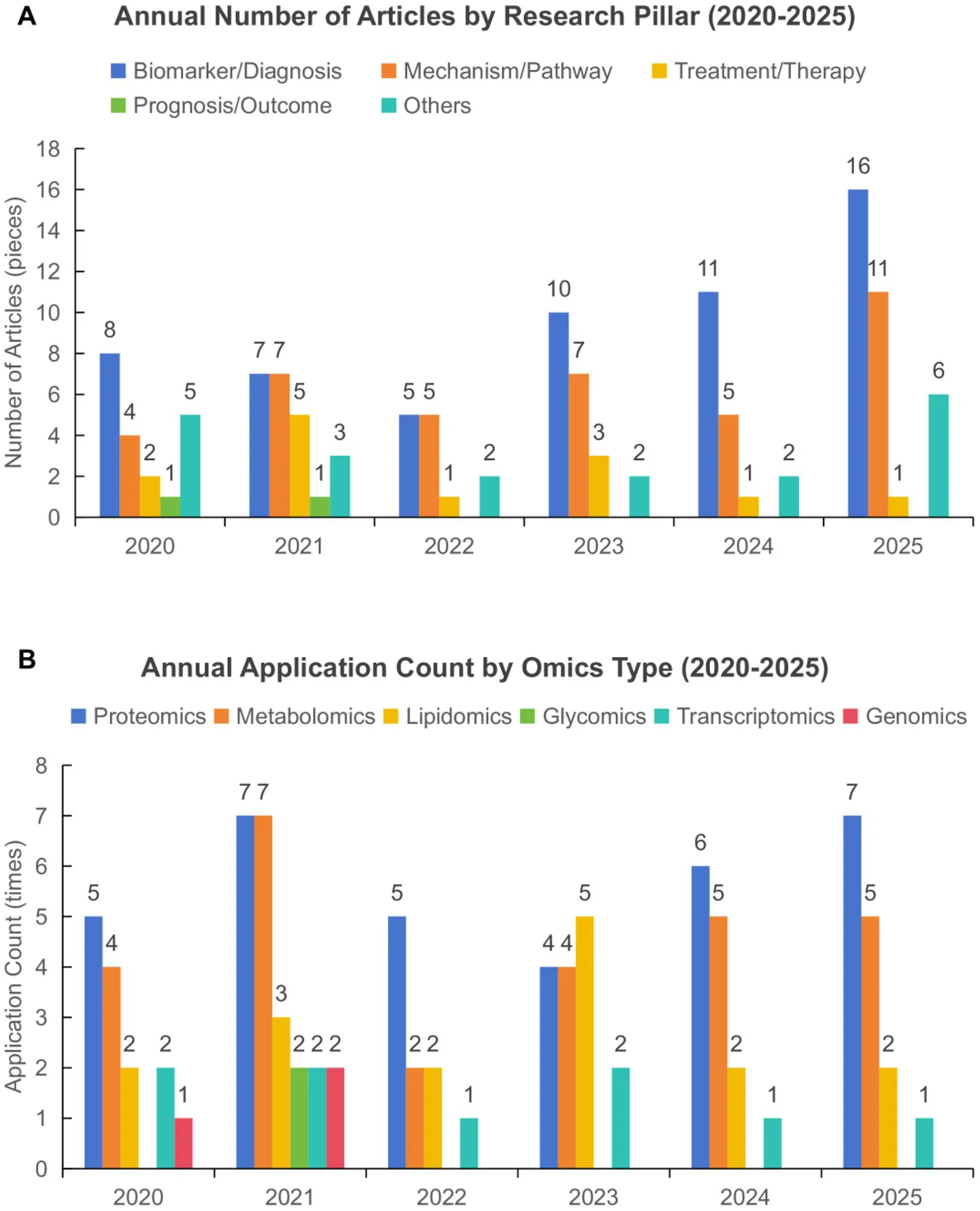
Annual publication trends of PD omics studies by research focus and omics type (2020–2025). **(A)** Annual number of articles categorized by research pillar: Biomarker/Diagnosis, Mechanism/Pathway, Treatment/Therapy, Prognosis/Outcome, and Others. A clear upward trend is observed across the period, with Biomarker/Diagnosis research (peaking at 16 articles in 2025) and Mechanism/Pathway studies (11 articles in 2025) showing the most substantial growth, reflecting increasing focus on translational and mechanistic PD research. **(B)** Annual application count of distinct omics types: Proteomics, Metabolomics, Lipidomics, Glycomics, Transcriptomics, and Genomics. Proteomics emerged as the most frequently applied omics approach (e.g., 7 applications in 2021 and 2025), followed by Metabolomics, highlighting their central role in PD omics investigations. Data are derived from the 127 articles included in this review, illustrating temporal shifts in research priorities and the adoption of omics methodologies.

From 2020–2025 (Figure 2), although PD omics research has encompassed diverse sample types and technical approaches, it has consistently centered on core objectives including elucidating pathogenic mechanisms, identifying clinical biomarkers, and discovering interventional targets, thereby forming a well-defined primary research agenda.

### 3.1 Elucidate the core molecular mechanisms underlying PD pathogenesis

#### 3.1.1 Mitochondrial dysfunction: from ‘metabolic phenomenon’ to ‘elucidation of the causal chain’

Mitochondrial dysfunction represents the most conserved pathological feature of PD and research has achieved the leap from identifying such abnormalities to defining their regulatory mechanisms. Multiple studies have illustrated this progress: Plum et al. (2020) performed synaptosomal proteomic analysis of the substantia nigra pars compacta (SNpc) using samples from 5 patients with advanced PD and 5 healthy controls, identifying the downregulated expression of 14 mitochondrial ribosomal proteins in PD patients and thereby indicating impaired mitochondrial translational function (Plum et al., 2020). In the same year, Bus et al. (2020) integrated transcriptomic and proteomic analyses to investigate PINK1-deficient dopaminergic neurons, confirming a direct association between mitochondrial metabolic abnormalities and dopamine synthesis dysregulation. Toomey et al. (2022) performed proteomic analysis of 9 brain regions and further established that mitochondrial dysfunction precedes *α*-Synuclein aggregation and neuronal loss, acting as a key early driver of PD (Toomey et al., 2022). Jang et al. (2023) conducted proteomic analyses and found that the magnitude of downregulation of mitochondrial ribosomal proteins was the most pronounced among all differentially expressed proteins, which was associated with the dysregulation of downstream pathways including RNA splicing and complement activation (Jang et al., 2023). De Mello et al. (2025) integrated metabolomic analyses of PARK7-knockout mice and fibroblasts derived from patients with early-onset familial PD, and unveiled that a high-sugar and high-fat diet exacerbates the selective atrophy of glycolytic muscle fibers by promoting the accumulation of advanced glycation end products (AGEs), thereby, the interplay between genetic susceptibility (PARK7 mutation) and environmental exposure (high-sugar and high-fat diet) leads to widespread AGEs accumulation, which ultimately induces PD-related phenotypes such as muscle atrophy. This process was independent of the impairment of mitochondrial oxidative phosphorylation and the reprogramming of energy metabolism (De Mello et al., 2025).

Collectively, these studies reveal that mitochondrial dysfunction acts as an early driver of PD, featuring altered mitochondrial ribosomal proteins and disrupted downstream pathways, while genetic mutation and high-energy diet jointly induce PD-related phenotypes independent of mitochondrial energy metabolism.

#### 3.1.2 Lipid metabolic dysregulation: from ‘single lipid abnormality’ to ‘network regulation’

Lipid metabolic dysregulation pervades the entire course of PD pathogenesis and research over the past years has gradually advanced the understanding from ‘Specific Lipids’ to the integrated ‘lipid-protein-inflammation’ regulatory network. Many studies have illustrated this progress: Ventura et al. (2020) performed HILIC-ESI-FTMS-based lipidomic analysis on dermal fibroblasts from PD patients at Hoehn-Yahr stage 5, and identified a significant downregulation of phosphatidylinositol containing the fatty acid chain C20:3(PI 38:3) (Ventura et al., 2020). In the same year, Hu et al. (2020) integrated proteomic and metabolomic analyses, confirming the activation of sphingolipid metabolism and decreased apolipoprotein levels in the plasma of PD patients, which indicated impaired lipid transport function. Fernández-Irigoyen et al. (2021) conducted lipidomic analyses on 30 post-mortem CSF samples and identified that in PD neuropathological staging, sphingomyelin was markedly elevated in Lewy Body Disease Early stage (LBDL) (early stage), phosphatidylcholine was markedly elevated in both LBDL and Lewy Body Disease Extended stage (mid-stage), whereas Lewy Body Disease Neocortical stage (late stage) was characterized by a prominent increase in polyunsaturated fatty acids (PUFAs) and triacylglycerols (TAG) (Fernández-Irigoyen et al., 2021). Galvagnion et al. (2022) performed lipidomic analyses on fibroblasts from GBA-mutant PD patients, establishing a direct correlation between aberrant sphingolipid composition and reduced glucocerebrosidase activity, and that aberrant lipid extracts accelerated *α*-Synuclein aggregation (Galvagnion et al., 2022).

From 2023 to 2025, research has further established associations between lipid metabolic dysregulation and clinical phenotypes: García-Fernández et al. (2023) conducted investigations using MPTP-treated cynomolgus monkey models of PD and found that in the putamen—a PD-relevant brain region—lipidomic analyses revealed a significant reduction in the content of phosphatidylserine (38,1), alongside a marked increase in the density of binding sites for mitochondrial electron transport chain Complex I. Panunggal et al. (2025) then confirmed that treadmill exercise ameliorates motor function by restoring the levels of phosphatidylethanolamine in the brain and lysophosphatidylethanolamine in the muscle of PD rats, thereby providing empirical evidence for the modifiability of lipid metabolism in PD.

Collectively, these studies reveal distinct lipid abnormalities in PD patients at different disease stages. Abnormal sphingolipid metabolism and impaired lipid transport are prevalent in PD, while GBA mutation-induced lipid dysregulation can further drive *α*-Synuclein aggregation. Region-specific lipid changes are closely linked to mitochondrial function in the brain. Moreover, lipid metabolic disorders are closely correlated with PD clinical manifestations, and exercise intervention can effectively regulate lipid profiles to alleviate motor impairments.

#### 3.1.3 *α*-Synuclein pathology: from ‘aggregation association’ to ‘structure–function regulation’

Aberrant aggregation of α-Synuclein constitutes the core pathological hallmark of PD, and research in this field over the past years has focused on its post-translational modifications, interacting proteins, and the underlying regulatory mechanisms. Multiple relevant studies have confirmed this point: Van Diggelen et al. (2020) performed proteomic analysis on rat primary hippocampal neurons, identifying 29 proteins that interact with DHA-stabilized *α*-Synuclein oligomers and predicting their key binding sites (Van Diggelen et al., 2020). Zhu et al. (2022) performed CSF proteomic analysis based on the Swedish NYPUM cohort and found that the levels of self-aggregating neuropeptides were significantly decreased in the CSF of PD patients; these self-aggregating neuropeptides are colocalized with *α*-Synuclein in neuronal dense-core vesicles, and one of the core functions of α-Synuclein is to regulate the secretory process of such vesicles. Thus, aberrant levels of self-aggregating neuropeptides may interfere with the normal packaging and secretion of α-Synuclein, thereby contributing to the pathological process of its aberrant aggregation (Zhu et al., 2022). Zhang et al. (2023) conducted proteomic analysis of brain tissue and identified post-translational modifications of *α*-Synuclein including phosphorylation at Y39 and acetylation at K21/K23; among these, phosphorylation at Y39 potently blocked the propagation of brain-derived pathological *α*-Synuclein, thereby providing a key target for modulating its aberrant aggregation (Zhang et al., 2023). Tripathi et al. (2024) constructed a semi-automated AlphaLarge meta-predictor based on proteomics, which elucidated the interaction structure between Synphilin-1—a large protein containing 919 residues—and A53T/A30P/E46K mutant α-Synuclein. The study revealed that the structural disorder of mutant α-Synuclein was slightly elevated, and its binding affinity to Synphilin-1 was significantly enhanced; the core of their interaction was localized to the C-terminus of Synphilin-1, which promoted the formation of aberrant aggregates into Lewy bodies via an increased number of residue pairs. These findings provide a critical structural basis for the targeted modulation of their aberrant co-aggregation and the intervention in the pathological progression of PD (Tripathi et al., 2024).

These multi-omics studies uncover the protein interaction network, regulatory modifications and structural mechanisms underlying α-Synuclein aggregation. They identify key interacting proteins and neuropeptides that interfere with *α*-Synuclein packaging and secretion. Specific post-translational modifications are verified to regulate the spread of pathological α-Synuclein. In addition, mutant α-Synuclein exhibits enhanced binding affinity with Synphilin-1, facilitating abnormal aggregate formation, which offers important structural targets for intervening PD progression.

#### 3.1.4 Gut-brain axis dysregulation: from ‘microbiota differences’ to ‘metabolite-mediated regulatory cascade’

The gut-brain axis constitutes an emerging research direction in PD studies, and research from 2021 to 2025 has gradually elucidated the regulatory pathway of the ‘gut microbiota-metabolite-brain pathology’ axis. Many relevant studies have confirmed this point: Rosario et al. (2021) integrated microbiomic and metabolomic analyses and found that the mucin-degrading capacity of the gut microbiota in PD patients was positively correlated with their Unified Parkinson’s Disease Rating Scale (UPDRS) scores. Furthermore, dysregulated microbial metabolism led to folate deficiency and elevated homocysteine levels, which indirectly promoted neuroinflammation in the brain (Rosario et al., 2021). Singh et al. (2023) conducted multi-omic analyses on transgenic PD rats, which revealed that age-related gut dysbiosis was accompanied by elevated succinate levels, and that antibiotic treatment reduced *α*-Synuclein levels in the forebrain (Singh et al., 2023). Chen et al. (2025) performed a validation study using double cohorts from Taiwan (63 PD patients + 54 controls) and Malaysia (36 PD patients + 20 controls), finding that the levels of caffeine and its metabolites were significantly decreased in PD patients and were negatively correlated with 56 species of enriched microbiota. This indicated that the gut microbiota modulates caffeine metabolism to exert an influence on motor symptoms of PD (Chen et al., 2025). These studies demonstrate that gut microbiota abnormalities are closely linked to PD severity and neuroinflammation. Age-related gut dysbiosis can elevate succinate levels and regulate cerebral α-Synuclein accumulation. Cross-regional cohort validation further confirms that gut microbiota affects PD motor symptoms by modulating caffeine metabolism, systematically clarifying the regulatory cascade of the gut microbiota-metabolite-brain pathology axis in PD pathogenesis.

Key representative multi-omics mechanistic studies are systematically summarized in Supplementary Table S2, including omics layers, sample types, sample size, core pathways and integration strategies.

### 3.2 Screening for clinically applicable biomarkers

#### 3.2.1 Diagnostic biomarkers: from ‘invasive samples’ to ‘non-invasive panels’

Diagnostic biomarkers need to feature high sensitivity, non-invasiveness and accessibility, and research conducted in recent years has gradually broadened available sample types and lifted diagnostic efficacy. Below are several typical examples for reference: Anjo et al. (2020) performed plasma proteomic analysis and found that a linear discriminant model constructed from the combination of Clusterin and VPS35 could distinguish PD patients from healthy controls, and VPS35 was the first PD-associated protein reported in plasma (Anjo et al., 2020). Rotunno et al. (2020), in turn, conducted CSF proteomic analysis and identified 13 consistently differential proteins, laying the foundational groundwork for CSF-based diagnostic biomarkers. Sinclair et al. (2021) conducted metabolomic analyses on 274 sebum samples and identified 10 lipid metabolites that could serve as potential biomarkers for PD, with sebum samples exhibiting the advantages of being non-invasive and easily accessible (Sinclair et al., 2021). Hirschberg et al. (2023) performed proteomic analyses on cerebrospinal fluid-derived extracellular vesicles and unpurified CSF, enrolling samples from patients with PD, its comorbid subtypes including Alzheimer’s disease (AD) and dementia with Lewy bodies (DLB), as well as healthy controls, and identified 13 PD-specific biomarkers in CSF-derived samples (Hirschberg et al., 2023). Abdi et al. (2023) conducted serum proteomic studies on the DeNoPa cohort; via limma differential expression analysis and Boruta feature selection analysis, they jointly confirmed 14 stable potential diagnostic biomarkers for PD. Meanwhile, they found that the level of total serum *α*-Synuclein in PD patients was significantly higher than that in healthy controls, and this difference remained statistically significant after excluding hemolyzed samples and outliers. Hällqvist et al. (2024) detected plasma proteins using targeted multiple mass spectrometry, and the constructed 8-protein panel achieved 100% identification of PD patients and 79% identification of iRBD individuals 7 years prior to symptom onset, enabling early disease warning (Hällqvist et al., 2024). Liu et al. (2025) conducted plasma metabolomic analysis and found that a predictive model composed of a combination of 5 metabolites achieved an AUC of 0.94; these metabolites were positively correlated with intestinal permeability markers, thus integrating diagnostic value with mechanistic relevance (Liu et al., 2025).

These studies reveal that PD diagnostic biomarkers have evolved toward high sensitivity, non-invasiveness and easy accessibility. Research has expanded sample types from traditional CSF to plasma, sebum and serum, shifting from single molecular markers to multi-protein and multi-metabolite panels. These signatures can accurately distinguish PD patients, identify prodromal individuals years before symptom onset, and correlate with intestinal and pathological mechanisms, greatly improving diagnostic performance.

We further note that *α*-synuclein seed amplification assays (SAA), including real-time quaking-induced conversion (RT-QuIC) and protein misfolding cyclic amplification (PMCA), have emerged as one of the most transformative biomarker advances from 2023 to 2025. Unlike routine omics biomarkers that merely reflect molecular abundance changes, SAA specifically detects prion-like pathological α-synuclein seeding activity, achieving high diagnostic accuracy with pooled sensitivity over 88% and specificity above 95% in cerebrospinal fluid specimens (Grossauer et al., 2023). Validated in minimally invasive samples including skin biopsy and peripheral blood, the α-synuclein seed amplification assay (SAA) shows distinct strengths in identifying preclinical individuals with isolated REM sleep behavior disorder (iRBD), differentiating neuronal α-synuclein disease from multiple system atrophy (MSA), and facilitating biological staging and stratification of synucleinopathy research cohorts (Simuni et al., 2024). As a functionally pathological detection approach complementary to multi-omics molecular panels, SAA has become an indispensable tool for early diagnosis and precise stratification in contemporary PD biomarker research.

#### 3.2.2 Subtyping biomarkers: from ‘genetic subtyping’ to ‘clinical phenotype correlation’

Subtyping biomarkers are designed to distinguish different subtypes of PD, thereby providing the theoretical and experimental basis for precision diagnosis and treatment of the disease. Karayel et al. (2020) performed proteomic analysis on neutrophils and found that the pRab10 levels in patients with LRRK2 G2019S and VPS35 D620N mutations were 1.9-fold and 3.7-fold higher than those in healthy controls, respectively, which could effectively distinguish the genetic subtypes of PD (Karayel et al., 2020). Several representative examples are presented as follows: Zhu et al. (2022) found that the levels of CLU and FBLN1 in the CSF of tremor-dominant (TD) PD patients were significantly higher than those in patients with the postural instability and gait difficulty (PIGD) subtype (Zhu et al., 2022). Zafar et al. (2022) conducted CSF proteomic analysis on patients with GBA-mutant PD (22 cases) and idiopathic PD (7 cases), and identified 20 uniquely differential proteins specific to GBA-mutant PD(PD_GBA), which indicated more severe synaptic damage and oxidative stress in these patients. Maple-Grødem et al. (2023) conducted CSF proteomic analysis on the ParkWest cohort and found that tau protein levels were positively correlated with the rate of cognitive decline at 10 years post-PD diagnosis, and could predict the conversion of cognitive subtypes of PD (Maple-Grødem et al., 2023). Tkachenko et al. (2025) performed plasma lipidomic analysis and revealed that HexCer levels were higher in PD patients than in those with AD and healthy controls, while their LPC levels were lower than those in healthy controls (Tkachenko et al., 2025).

These findings demonstrate that subtyping biomarkers can effectively differentiate PD genetic subtypes, motor phenotypes, and cognitive progression. Multiple proteomic and lipidomic signatures in CSF and plasma enable the distinction between mutation-related PD, idiopathic PD, and other neurodegenerative disorders. Such molecular markers also reflect subtype-specific pathological changes and predict long-term cognitive deterioration, laying a solid foundation for precise PD classification and individualized clinical management.

#### 3.2.3 Therapeutic monitoring biomarkers: from ‘target evaluation’ to ‘clinical response prediction’

Therapeutic monitoring biomarkers are used to evaluate the pharmacological efficacy of drugs and interventional effects of therapeutic measures, and research over the past 6 years has gradually expanded from basic target assessment to clinical response prediction. The following cases serve as typical references: Karayel et al. (2020) found that neutrophil pRab10 levels could be used to evaluate the efficacy of LRRK2 inhibitors, with pRab10 levels decreasing by more than 30% after treatment with the inhibitors (Karayel et al., 2020). Yoo et al. (2020) performed proteomic analysis and found that the expression of proteins associated with synaptic vesicle trafficking was upregulated, which could serve as a molecular indicator for the efficacy of Entacapone. O’Bryant et al. (2022) analyzed plasma proteomic data from the DATATOP clinical trial involving 520 PD patients, and the constructed inflammatory protein predictive model could distinguish the treatment response status of patients in the selegiline and *α*-tocopherol groups with high accuracy (O’Bryant et al., 2022). Rutledge et al. (2024) analyzed samples from the PPMI cohort using three techniques: Olink, Soma-Scan and LC–MS/MS and found that dopa decarboxylase (DDC) levels in CSF and urine were elevated in untreated PD patients, prodromal PD individuals, and LRRK2/GBA carriers alike, and were positively correlated with UPDRS III scores, enabling the monitoring of disease progression (Rutledge et al., 2024). Gątarek et al. (2025) conducted urinary metabolomic analysis and found that homovanillic acid levels could serve as a biomarker for therapeutic monitoring. Meanwhile, succinate levels were negatively correlated with H-Y stage, whereas TMAO levels were positively correlated with it, which integrates the evaluation of therapeutic efficacy with the assessment of disease severity.

These studies show that therapeutic monitoring biomarkers have evolved from basic target evaluation to accurate clinical response prediction. Molecular indicators such as pRab10 and synaptic vesicle-related proteins can reflect the efficacy of anti-PD drugs. Plasma inflammatory signatures, CSF and urine DDC levels are capable of tracking disease progression and treatment response. Urinary metabolites can also mirror PD severity and clinical staging, providing reliable molecular references for evaluating therapeutic effects and guiding clinical intervention strategies.

### 3.3 Identifying precision intervention targets

#### 3.3.1 Pathway targets: from ‘single pathway’ to ‘cross-regulation’

The validation and in-depth investigation of core pathway targets form the foundation of intervention research, and research over the past 6 years has focused on pathways including LRRK2-Rab, PI3K/AKT, and Nrf2/HO-1. Multiple relevant studies have confirmed this point: Bonet-Ponce et al. (2020) performed proteomic analysis of astrocytes from LRRK2-knockout mice and found that LRRK2 could mediate the lysosomal injury repair (LYTL) process by phosphorylating RAB35/RAB10 to recruit JIP4, and pathogenic mutations potentiated this process (Bonet-Ponce et al., 2020). In the same year, Karayel et al. (2020) confirmed that aberrations in the LRRK2-Rab pathway were associated with patient stratification in PD. Li et al. (2023) performed multi-omics analysis on MPTP-induced Parkinson’s disease (PD) mice and found that(−)-Clausenamide could bind to the Ser663 site of ALOX5, inhibit its nuclear translocation and activation, reduce lipid peroxidation, and protect dopaminergic neurons against ferroptosis (Li et al., 2023). In 2025, an integrative MMT_CA model combining multi-omics and DaTscan imaging achieved high accuracy for PD diagnosis (see Section 4.2.3) (Zhou et al., 2025). Li et al. (2025), in turn, confirmed that the downregulation of the rate-limiting enzyme involved in glutathione synthesis contributes to the pathogenesis of PD by exacerbating oxidative stress.

These omics studies highlight LRRK2-Rab, PI3K/AKT and other key signaling pathways as core therapeutic targets. LRRK2 mutations modulate lysosomal repair processes and correlate with PD patient heterogeneity. Natural compounds can target ALOX5 to suppress lipid peroxidation and protect dopaminergic neurons. Dysregulation of glutathione synthesis also aggravates oxidative stress to drive PD progression, while multi-omics combined imaging models further support pathological mechanism exploration and precise diagnosis.

#### 3.3.2 Molecular targets: from ‘basic validation’ to ‘preclinical exploration’

The clinical translational potential of molecular targets is the focus of research, and research over the past 6 years has centered on druggable targets such as GCase, GPNMB, and ALOX5.

In 2022, this work confirmed that abnormal sphingolipid composition in GBA-mutant fibroblasts accelerates *α*-synuclein aggregation and impairs GCase activity (see Section 3.1.2) (Galvagnion et al., 2022). Bogetofte et al. (2023) performed proteomic analysis on iPSC-derived dopaminergic neurons from PD patients with the GBA-N370S mutation and found that small-molecule chaperones of GCase could rescue neurite outgrowth defects (Zhang et al., 2023). Ge et al. (2023), in turn, identified GPNMB as the most promising dual target (brain + blood) for PD through multi-cohort validation. Cortés et al. (2025) performed phosphoproteomic analysis on the Spanish LRRK2 cohort (174 samples) and found that pSer106 RAB12 is a blood-based biomarker for G2019S-driven PD (Cortés et al., 2025).

These studies focus on druggable molecular targets including GCase, GPNMB and RAB12. GCase dysfunction induced by GBA mutations aggravates pathological aggregation of *α*-Synuclein, and small-molecule chaperones can effectively rescue neuronal defects. GPNMB is validated as a promising dual target detectable in both brain and blood, while phosphorylated RAB12 serves as a specific peripheral biomarker for LRRK2-related PD, offering valuable candidates for clinical translation and targeted therapy.

#### 3.3.3 Microbial targets: from ‘gut microbiota modulation’ to ‘functional validation’

Gut microbiota represent emerging targets for PD intervention, and research over the past 6 years has expanded from the characterization of gut microbiota composition to functional validation. Tan et al. (2021) performed microbiomic and metabolomic analyses on 104 PD patients and 96 healthy controls, and found that short-chain fatty acid (SCFA) levels were decreased in PD patients and were negatively correlated with cognitive function. Singh et al. (2023) confirmed that antibiotic treatment could alter the gut metabolites of PD rats and reduce α-Synuclein levels in the forebrain (Singh et al., 2023). Ovalle et al. (2025) designed a microbial consortium containing Lactobacillus, which could ameliorate motor deficits in PD Drosophila models (Ovalle et al., 2025). Chen et al. (2025), in turn, found that the gut microbiota associated with caffeine metabolism was linked to paraxanthine reduction and PD.

These studies indicate that gut microbiota have become novel intervention targets for PD, with research advancing from compositional description to functional verification. Reduced short-chain fatty acids in PD patients are closely associated with cognitive impairment. Antibiotic intervention can reshape gut metabolites and lower cerebral *α*-Synuclein levels. Rational microbial supplementation alleviates motor deficits in PD models, and gut microbiota also participates in PD pathogenesis by regulating caffeine metabolism, providing solid evidence for gut-targeted therapeutic strategies.

Representative druggable molecular and pathway targets are summarized in Supplementary Table S4.

## 4 Evolutionary trajectory of omics research techniques for Parkinson’s disease (2020–2025)

The iteration of omics technologies constitutes the core driving force behind the deepening of research objectives for PD. From 2020–2025, technical approaches have evolved from basic single-omics detection to systems-level multi-omics modeling, and supporting technical systems have expanded from small-scale, single-center samples to large-scale, multi-center big data, thus forging a complete technical pipeline of detection-integration-validation.

### 4.1 Single-omics technologies: from ‘basic quantification’ to ‘precision targeting’

#### 4.1.1 Proteomics: from ‘traditional mass spectrometry’ to ‘highly specific targeted technologies’

Advancements in proteomic technologies focus on improving detection of low-abundance proteins, refining post-translational modification characterization, and broadening available sample types. Plum et al. (2020) analyzed synaptosomal proteins in the SN by LC–MS/MS and identified only hundreds of proteins (Plum et al., 2020). Wen et al. (2021) investigated the secretory granules of MPP^+^-treated SH-SY5Y cells using LC–MS/MS combined with label-free quantitative proteomic analysis, and identified a total of 4,249 proteins, among which 536 were significantly differentially expressed proteins (Wen et al., 2021). In 2022, the targeted LC–MS/MS MRM technology was applied for biomarker validation; for example, this study reported altered CSF CLU and FBLN1 levels distinguishing tremor-dominant and PIGD PD subtypes, which was further validated by targeted MRM proteomicssee (Section 3.2.2) (Zhu et al., 2022). Salim et al. (2023) analyzed α-Synuclein in red blood cells using this technology, with the limit of detection (LOD) reaching 0.02 μg/mL, outperforming CE-MS and direct MS (Salim et al., 2023). In 2024–2025, structural proteomics and proximity proteomics technologies emerged; Tripathi et al. (2024) developed the AlphaLarge meta-predictor and elucidated the interaction structure between α-Synuclein and Synphilin-1.

#### 4.1.2 Lipidomics: from ‘single-class detection’ to ‘global profiling’

The evolution of lipidomic technologies has focused on ‘expanding lipid coverage, enhancing structural resolution capability, and linking to functional validation’. In 2020, research centered on single lipid classes; for example, Xicoy et al. (2020) analyzed lipids in 6-OHDA-treated SH-SY5Y cells via LC–MS and identified the protective effect of cholesterol, albeit with the limited applicability of the employed cellular model.

UPLC–MS/MS was introduced for global lipid profiling; Fernández-Irigoyen et al. (2021) analyzed multiple lipid classes including SM, PC, and PE in CSF via UPLC–MS/MS (Fernández-Irigoyen et al., 2021). MALDI-MS was applied for tissue lipid imaging; García-Fernández et al. (2023) analyzed lipids in brain regions of PD models in cynomolgus monkeys via MALDI-MS and found that changes in phosphatidylserine (38,1) in the olfactory bulb and putamen were associated with the activity of electron transport chain (ETC) complexes (García-Fernández et al., 2023). 4D glycosphingolipidomics technology enabled refined structural characterization; Bindila et al. (2025) analyzed serum samples via this technology and found that 41 species of gangliosides and neolacto-series glycosphingolipids could distinguish PD patients from healthy controls, and 14 of these lipids could differentiate between gender subgroups of PD, yet the technology failed to provide structural information such as glycosidic bond types (Vo et al., 2025).

#### 4.1.3 Metabolomics: from ‘targeted detection’ to ‘multi-technology integration’

Advancements in metabolomics are geared toward expanding metabolite coverage, validating findings across multiple analytical platforms, and establishing mechanistic links with microbial metabolism. Crotty et al. (2020) analyzed the LCC cohort via liquid chromatography-mass spectrometry (LC/MS) and found that caffeine and its metabolites were associated with PD resistance in LRRK2 mutation carriers.

Sinclair et al. (2021) analyzed sebum samples via LC–MS and found that abnormal lipid metabolism was associated with PD. Talavera Andújar et al. (2022) optimized the chemoinformatics workflow, analyzed plasma and fecal samples via LC–MS/MS, and identified an association between gut microbial metabolites and PD. UHPLC–MS/MS enabled high-resolution detection; LeWitt et al. (2023) analyzed CSF samples via UHPLC–MS/MS and found that a panel consisting of 23 compounds achieved an AUC of 0.897 for PD prediction, and the polyamine pathway was involved in neurodegeneration (LeWitt et al., 2023). In 2024–2025, multi-technology integration allowed the coverage of multiple metabolite classes; Bobrowska-Korczaka et al. combined GC–MS with LC–MS/MS to analyze urinary metabolites and identified associations between succinic acid, HVA, TMAO and PD (Gątarek et al., 2025).

### 4.2 Multi-omics integration technologies: from ‘simple coupling’ to ‘systems modeling’

#### 4.2.1 Multi-omics technologies: integration of two omics panels for the validation of a single pathway

From 2020 to 2021, multi-omics studies predominantly featured the combinations of proteomics plus metabolomics and proteomics plus transcriptomics, with the aim of verifying the correlation of a single pathway. It is noteworthy that most two-omics combinations in this period belonged to simple data juxtaposition rather than systematic joint modeling, which were not genuine integrative multi-omics in the strict sense. Hu et al. (2020) integrated proteomics and metabolomics to analyze the plasma of PD patients and identified dysregulated lipid metabolism, yet no pathway network analysis was performed; Bus et al. (2020) integrated transcriptomics, proteomics and metabolomics and found that PINK1-deficient dopaminergic neurons exhibited dysregulation in mitochondrial metabolism and dopamine metabolism, with the study only focusing on a single gene deficiency. Zhang et al. (2021) integrated proteomics and metabolomics to analyze serum from PD patients with cognitive impairment, and found that LPC (18,1) was negatively correlated with cognitive levels, alongside abnormalities in the inflammatory-lipid metabolic network (Zhang et al., 2021); Niimi et al. (2021) integrated metabolomics and proteomics (focusing on CSF C5a), and identified that the level of C5a in the CSF of PD patients was downregulated, and glucosylceramide d18:1/C23:0 was positively correlated with C5a, yet the underlying mechanism was not further investigated.

#### 4.2.2 Multi-omics technologies: integration of three omics panels for the exploration of pathway networks

From 2022 to 2023, multi-omics studies expanded to the “three-omics panel” strategy and introduced basic algorithms (e.g., WGCNA, PPI networks), with the aim of exploring the crosstalk between pathways. Abdelmoaty et al. (2022) integrated transcriptomics (single-cell RNA sequencing) and proteomics to analyze monocytes from PD patients, and found that sargramostim treatment could activate anti-inflammatory, antioxidant, and autophagy-related pathways, with such activation being associated with UPDRS III scores (Abdelmoaty et al., 2022); Heremans et al. (2022) integrated metabolomics and proteomics and discovered that PARK7 could scavenge reactive intermediates formed by 1,3-BPG (a glycolytic metabolite), thereby preventing metabolite and protein damage. Li et al. (2023) integrated transcriptomics, proteomics, and metabolomics to analyze PD mice, and found that(−)-Clausenamide could protect dopaminergic neurons by inhibiting ALOX5 (Li et al., 2023); One multi-omics study revealed that age-related gut dysbiosis elevates succinate and promotes brain *α*-synuclein accumulation in PD models (see Section 3.1.4) (Singh et al., 2023).

#### 4.2.3 Multi-omics technologies: integration of multi-omics panels with AI algorithms for the construction of clinical models

In 2024–2025, multi-omics studies integrated genomics, transcriptomics, proteomics, metabolomics, and microbiomics, and incorporated AI algorithms (machine learning, Mendelian randomization, GCN models), with the aim of developing clinically applicable models for diagnosis, subtyping, and therapeutic efficacy prediction. Carrillo et al. (2024) first recruited 804 patients with PD and then selected a subset of these samples to conduct an integrated analysis of lipidomics and metabolomics. They found that the levels of diglucosyl ceramide (Hex2Cer, it contains two subtypes: lactosyl ceramide and digalactosyl ceramide) and sphinganine (SPB) were significantly elevated in the deep brain stimulation (DBS) group, both of which possessed high diagnostic efficacy; the area under the receiver operating characteristic (ROC) curve (AUC) of Hex2Cer reached 0.99 (It is worth noting that such near perfect diagnostic performance may only be applicable to small single center cohorts, with potential overfitting issues and sample specificity, and may not be generalizable to heterogeneous real-world Parkinson’s disease patient populations), and that of SPB was 0.91 (Carrillo et al., 2024). Zhou et al. (2025) integrated transcriptomics, proteomics, metabolomics, hematological information and DaTscan imaging to construct the MMT_CA model, which achieved a balanced accuracy of 97.7% for PD diagnosis, with DaTscan imaging identified as the greatest contributor (Zhou et al., 2025); Ryan et al. (2025) integrated genomics, transcriptomics (mRNA, miRNA), proteomics and epigenomics (DNAm) data to analyze the Parkinson’s Progression Markers Initiative (PPMI) cohort, and found that the combination of SNP and DNAm had the potential to serve as an early diagnostic tool for hereditary PD; longitudinal experiments demonstrated that the model trained on this combination exhibited optimal classification performance, achieving an accuracy of 0.974 and an F1 score of 0.965 in distinguishing PD, preclinical PD and healthy controls; Li et al. (2025) integrated proteomics and metabolomics and combined machine learning to identify that GCLC downregulation was associated with PD, and that GCLC overexpression could restore the function of dopaminergic neurons; Ovalle et al. (2025) integrated genomics, metabolomics and microbiomics, and found that microbial consortia could ameliorate motor deficits in PD Drosophila, with such amelioration being associated with abnormal brain metabolism.

Although these AI-integrated models achieve high diagnostic accuracy and AUC values, most are limited by relatively small single-center cohorts, which may raise the risk of overfitting. In addition, current models generally lack sufficient interpretability, making it a little difficult to clarify the internal biological logic behind omics feature classification.

### 4.3 Auxiliary technical support system: from ‘single-center small sample’ to ‘multi-center big data’

#### 4.3.1 Biobank: from ‘single-center small sample’ to ‘multi-center large cohort’

Development of modern biobanks centers on expanding sample cohorts, improving demographic diversity, and extending long-term longitudinal follow-up. In 2020, research was predominantly based on single-center small samples, with the sample size of most cohorts being fewer than 50 cases. For instance, Plum et al. (2020) only enrolled 10 SN tissue samples (5 PD cases and 5 controls); Karayel et al. (2020) included 24 neutrophil samples (14 controls and 10 PD cases), with no longitudinal follow-up conducted. In 2021, the integration of multi-center cohorts was initiated. For example, Shao et al. (2021) enrolled 3 independent cohorts (460 plasma samples); Avisar et al. included 149 patients with PD, and all participants in this cohort were non-carriers of SNCA, LRRK2 and GBA mutations. Of the 150 patients initially randomly selected, 1 was excluded due to missing UPDRS III scores and total UPDRS scores, with 149 patients finally included for analysis. In 2022, the cohort size was further expanded. Karayel et al. (2022) enrolled 215 samples (the HBS cohort plus the LCC cohort); Toomey et al. (2022) included Braak-staged PD patients and healthy controls, covering 9 brain regions. Longitudinal cohorts emerged for the first time. Abdi et al. (2023) enrolled the DeNoPa cohort (85 PD cases and 93 controls with an 8-year follow-up) (Abdi et al., 2023); Hirschberg et al. (2023) included 249 samples, which comprised subtypes such as PD-MCI and PDD. In 2024–2025, multi-center, multi-ethnic, large-sample cohorts became the mainstream. Imam et al. (2025) enrolled 23 contributing cohorts (ABC-PD, PPMI, BioFINDER, etc.); Ali et al. (2025) included 10,527 plasma samples from the GNPC cohort; Ryan et al. (2025) included 2,188 samples from the PPMI cohort, covering genetic forms of PD, idiopathic PD, and prodromal populations, with available longitudinal follow-up data.

#### 4.3.2 Data analysis tools: from ‘basic statistics’ to ‘AI algorithms’

Ongoing development of analytical tools prioritizes more efficient data integration, complex biological network mining, and systematic bias mitigation. In 2020, basic statistical methods (*t*-test, ANOVA, Pearson correlation analysis) dominated the field. For instance, Anjo et al. (2020) screened differential plasma proteins via the *t*-test; Yilmaz et al. (2020) controlled core confounding factors through study design and statistical tests when analyzing metabolomic differences using ANOVA, yet failed to incorporate these variables into the model for further optimization due to the lack of partial clinical information such as UPDRS scores.

Multivariate statistics (PCA, PLS-DA) were introduced into the field. For example, Shao et al. (2021) screened PD-associated differential metabolites via PLS-DA, and further combined binary logistic regression to construct a metabolite diagnostic model incorporating indole lactic acid and phenylacetylglutamine. Validated in three independent cohorts, this model exhibited excellent discriminative ability for distinguishing PD from healthy controls and PD from non-PD neurological disease controls (Shao et al., 2021); Rosario et al. (2021) analyzed the associations between gut microbiota and metabolites through metabolic modeling.

WGCNA and PPI networks were applied for pathway exploration. Oliveira et al. (2022) analyzed proteins in the RTN region of PD rats via WGCNA and identified abnormal purinergic signaling (Oliveira et al., 2022); Heremans et al. (2022) discovered that PARK7 prevented glycerate and phosphoglycerate modification of metabolites and proteins by scavenging reactive intermediates spontaneously formed by 1,3-bisphosphoglycerate (1,3-BPG), a glycolytic metabolite, thereby avoiding their damage.

Mendelian randomization, ExoView and Simoa were applied for causal validation, and Ge et al. (2023) confirmed GPNMB as a causal target of PD through Mendelian randomization (Ge et al., 2023). In 2024–2025, AI algorithms (machine learning, GCN models, MultiOmicsIntegrator, etc.) became the mainstream. Zhou et al. (2025) integrated multi-omics data via machine learning to construct a diagnostic model with high accuracy; A GCN-based integrative model enabled longitudinal stratification of PD, prodromal PD and healthy controls using PPMI cohort multi-omics data (see Section 4.2.3) (Ryan et al., 2025); Pasat et al. (2025) integrated transcriptomics and proteomics through MultiOmicsIntegrator and identified an association between the IRE1-RIDD pathway and PD.

#### 4.3.3 Validation system: from ‘no validation’ to ‘discovery-validation-follow-up closed loop’

Advancements of the validation system focuses on improving the reliability of findings, ensuring clinical applicability, and monitoring long-term stability. In 2020, most studies only established discovery cohorts without independent validation. For instance, Plum et al. (2020) did not validate the differential synaptic proteins in the SN; Barkovits et al. (2020) validated the impact of blood contamination on *α*-Synuclein detection and found that α-Synuclein levels increased significantly with the aggravation of blood contamination—ELISA quantification exhibited biases when blood contamination was ≥0.01%, whereas mass spectrometry (MS) quantification was markedly affected when blood contamination reached ≥0.1%.

The establishment of independent validation cohorts was initiated. For example, Shao et al. (2021) established three independent validation cohorts to validate the metabolite panel (Shao et al., 2021); Avisar et al. (2021) recommended validating the lipid biomarkers associated with PD severity identified by their team in larger-scale cohorts that included fasting participants and longitudinal data. In 2022, Karayel et al. performed proteomic analysis of CSF using data-independent acquisition liquid chromatography–tandem mass spectrometry (DIA-MS) technology, and found that the expression of CD44 was significantly elevated in the CSF of PD patients. This protein was stably quantified in two independent validation cohorts, serving as a potential candidate biomarker for distinguishing PD patients from healthy controls; Galvagnion et al. (2022) validated glucocerebrosidase (GCase) activity via the fluorescent substrate assay, using 4-methylumbelliferyl-*β*-D-glucopyranoside as the substrate, and quantitatively analyzed enzyme activity by detecting the fluorescence intensity of enzymatic reaction products.

In 2023–2025, the Discovery-Validation-Longitudinal Follow-up closed loop was fully established. Abdi et al. (2023) validated the stability of serum biomarkers through an 8-year follow-up; Hällqvist et al. (2024) verified the early warning value of biomarkers using a longitudinal cohort of isolated rapid eye movement sleep behavior disorder (iRBD); Ryan et al. (2025) confirmed the long-term accuracy of diagnostic models via longitudinal data from the PPMI cohort.

## 5 Common findings and evolutionary characteristics of PD omics research (2020–2025)

PD omics research from 2020–2025 has yielded a series of common findings in three core directions: mechanisms, biomarkers and targets, and exhibited the evolutionary characteristics of ‘from phenomena to essence, from basic research to clinical application, and from a single perspective to a systematic approach’. Over the six-year research period, repeated validation has clarified the core pathogenic mechanisms of PD onset, gradually uncovered the crosstalk among these mechanisms, and thus formed a comprehensive pathological network.

### 5.1 Common validation and deepening of core mechanisms: from ‘single abnormality’ to ‘network regulation’

#### 5.1.1 Crosstalk among mechanisms: forming the ‘genetics-environment-pathology’ chain

##### 5.1.1.1 Mitochondria and lipid metabolism

In 2022, this work confirmed that abnormal sphingolipid composition in GBA-mutant fibroblasts accelerates *α*-synuclein aggregation and impairs GCase activity (see Section 3.1.2) (Galvagnion et al., 2022). García-Fernández et al. (2023) confirmed in MPTP-induced Parkinson’s disease monkey models that alterations in specific phosphatidylserine subtypes had brain region-specific correlations with the activity of mitochondrial electron transport chain (ETC) complexes: in the core PD-affected brain regions including the globus pallidus, putamen, caudate nucleus and SN, the content of PS 38:1 was significantly decreased and showed a local negative correlation with the elevated activity of Complex II; in the cerebellum, a brain region relatively preserved in PD, the content of PS 38:1 was instead increased, and the direction of its correlation with ETC complex activity was opposite to that in the core brain regions (García-Fernández et al., 2023).

##### 5.1.1.2 α-Synuclein and mitochondria

Zhang et al. (2023) found that Y39 phosphorylation of α-Synuclein could reduce the amplification of pathological α-Synuclein derived from Lewy body disease and multiple system atrophy and decrease its seeding activity. This effect was conformation-dependent and exerted no significant influence on *in vitro* synthesized α-Synuclein preformed fibrils (PFFs) (Zhang et al., 2023). Tripathi et al. (2024) deciphered the interaction structure of Synphilin-1 with α-Synuclein carrying the A30P, A53T and E46K mutations via the self-developed AlphaLarge meta-predictor and found that this mutant complex exhibited stronger binding affinity, which could promote the formation of Lewy body-associated toxic aggregates. This finding provides a critical structural basis for targeted drug screening in PD (Tripathi et al., 2024).

##### 5.1.1.3 Gut-brain axis and inflammation

In 2023, a multi-omics study revealed that age-related gut dysbiosis elevates succinate and promotes brain α-synuclein accumulation in PD models (see Section 3.1.4). Chen et al. (2025) identified a significant negative correlation between specific gut microbiota enriched in Parkinson’s disease patients and plasma paraxanthine levels through a multicenter cohort study: paraxanthine levels in PD patients were significantly lower than those in healthy individuals, and paraxanthine levels were negatively correlated with the severity of PD motor symptoms, suggesting that such microbiota may interfere with caffeine metabolism to alter paraxanthine levels and thus be associated with the pathological progression of PD (Chen et al., 2025). De Mello et al. (2025) further integrated genetic and environmental factors and found that PARK7 mutation (genetics) combined with a high-sugar and high-fat diet (environment) could induce pathological phenotypes associated with early-onset Parkinson’s disease through extensive pathological accumulation of Advanced Glycation End Products (AGEs). This finding confirmed the association among diet, AGEs accumulation and the genetic background of PD, forming the ‘Genetics-Environment-Pathology’ regulatory chain (De Mello et al., 2025).

#### 5.1.2 Phase specificity of mechanisms: early onset driving and late-stage aggravation

*Early stage* (Preclinical Stage/Initial Diagnosis Stage): Mitochondrial dysfunction, gut microbiota dysbiosis and lipid metabolic disorder [Avisar et al. (2021) identified an elevated level of dhSM in patients with early-stage PD] are the key driving factors.

*Mid stage* (Motor Symptom Stage): α-Synuclein aggregation. Zhang et al. (2023) found that Y39 phosphorylation of soluble α-Synuclein regulates the amplification of pathological α-Synuclein, and this effect is conformation-dependent – it markedly represses the nucleation activity of Lewy body-derived LB- α-Synuclein, mildly inhibits the amplification of multiple system atrophy-derived GCI- α-Synuclein, and exerts no significant effect on in vitro synthesized α-Synuclein preformed fibrils and inflammatory response (Abdelmoaty et al. found that in PD patients following sargramostim treatment, anti-inflammatory-related proteins including HMOX1, TLR2, TLR8 and RELA in monocytes were significantly downregulated at 2 and 6 months post-treatment (Abdelmoaty et al., 2022), and these changes were associated with the improvement of motor function in patients) emerge as the major pathological features.

*Late stage* (Dementia Stage): This stage is characterized by the superimposition of multiple mechanisms. For instance, Kalecký et al. (2022) found that intracerebral unbound homocysteine in patients with dementia-type PD increased significantly following levodopa intake, accompanied by insufficient activation of cystathionine *β*-synthase (CBS)—a phenomenon that may be associated with carbidopa-induced vitamin B6 deficiency—and concurrent impairment of the remethylation pathway mediated by methionine synthase (MTR).

### 5.2 Biomarker iteration and optimization: from ‘candidate molecules’ to ‘clinical panels’

#### 5.2.1 Sensitivity and specificity: from ‘low efficacy’ to ‘high-efficacy panels’

In 2020, the mean AUC of the plasma Clusterin + VPS35 panel was approximately 0.821 (Anjo et al., 2020). In 2021, the multivariate diagnostic model based on sebum metabolites yielded the following AUC values for distinguishing Parkinson’s disease (PD): 0.779 (95% CI, 0.688–0.889) for drug-naïve patients and 0.806 (95% CI, 0.661–0.859) for medicated patients (Sinclair et al., 2021). In 2024, an 8-plasma protein panel achieved an AUC of 1.0 for PD identification (Hällqvist et al., 2024). In 2025, a 5-metabolite panel reached an AUC of 0.94 (Liu et al., 2025). Notably, most high-performance diagnostic models are established and validated on limited sample sizes, which may lead to overfitting and poor external generalization. Meanwhile, existing machine learning models still lack transparent interpretability for omics-based biomarker screening.

#### 5.2.2 Clinical applicability: from ‘invasive’ to ‘non-invasive and accessible’

The sample types of biomarkers have been progressively optimized toward non-invasive and easily accessible alternatives: in 2020, research primarily relied on invasive samples such as CSF (Rotunno et al., 2020)and brain tissue (Plum et al., 2020), resulting in low clinical applicability; In 2021, the sample spectrum expanded to sebum (Sinclair et al., 2021) and urine (Virreira Winter et al., 2021), yet sebum sample collection required specialized equipment; in 2023, studies focused on blood samples (Abdi et al., 2023), which facilitated convenient sample collection; from 2024 to 2025, researchers developed plasma- and urine-based biomarkers (plasma (Hällqvist et al., 2024); urine (Rutledge et al., 2024)), and the detection methods for these biomarkers were compatible with routine clinical platforms (e.g., LC–MS/MS, ELISA), achieving a substantial improvement in accessibility.

#### 5.2.3 Functional expansion: from ‘single diagnosis’ to ‘multidimensional application’

##### 5.2.3.1 Subtyping function

pRab10 distinguished genetic subtypes (Karayel et al., 2020) in 2020; CLU/FBLN1 distinguished motor subtypes (Zhu et al., 2022) in 2022; HexCer distinguished PD from AD (Tkachenko et al., 2025) in 2025.

##### 5.2.3.2 Prognostic function

Tau was associated with cognitive decline (Maple-Grødem et al., 2023) in 2023; methionine levels were associated with disease progression (Berezhnoy et al., 2025) in 2025.

##### 5.2.3.3 Therapeutic efficacy monitoring function

pRab10 was used to evaluate LRRK2 inhibitors (Karayel et al., 2020) in 2020; inflammatory proteins predicted treatment response (O’Bryant et al., 2022) in 2022; HVA was used to monitor the efficacy of levodopa (Gątarek et al., 2025) in 2025.

Detailed information of existing biomarker panels, diagnostic metrics and validation status are listed in Supplementary Table S3.

## 6 Common challenges of PD omics research

Nevertheless, relevant research still faces challenges such as limited sample sizes, cohort heterogeneity, inadequate technical standardization, and the translational gap. Addressing these issues requires the establishment of multicenter longitudinal cohorts, the advancement of high-sensitivity detection and AI-integrated technologies, and the promotion of precision subtyping, clinical validation of candidate targets, and exploration of non-pharmacological interventions. These efforts will translate research findings from the laboratory to the clinic, thereby facilitating the early diagnosis and precision therapy of PD.

Although PD omics research has achieved remarkable progress from 2020–2025, it still faces three major common challenges in terms of samples, technology and clinical translation. Targeted breakthroughs are therefore required in the future to facilitate its clinical application.

### 6.1 Sample challenges: insufficient quantity, quality, and diversity

Limited Sample Size: Most studies included fewer than 100 samples, with only a small number of studies (e.g., Ali et al., 2025; Imam et al., 2025) reaching the ten-thousand scale in 2025; small sample sizes result in insufficient statistical power. Insufficient Cohort Heterogeneity: Samples are mostly derived from Europe and North America, with a lack of data from Asian and African populations. For example, studies by Ge et al. (2023) and Gao C. et al. (2025) recruited primarily participants of European ancestry, limiting the generalizability of findings. In addition, inconsistent inter-center sample processing protocols lead to poor data consistency. Lack of Longitudinal Data: Only a few studies from 2023 to 2025 (e.g., Abdi et al., 2023; Hällqvist et al., 2024) have a follow-up duration of at least 5 years, making it impossible to evaluate the long-term stability of biomarkers and the long-term efficacy of target interventions.

### 6.2 Technical challenges: insufficient detection, integration, and standardization

Detection Limitations: Single-omics technologies still suffer from missed detections—for instance, proteomics struggles to detect low-abundance proteins (e.g., LRRK2) and hydrophobic transmembrane proteins; lipidomics fails to provide complete structural information (e.g., glycosidic bond types (Vo et al., 2025)); and metabolomics is susceptible to matrix interference, among other issues. Immature Integration Algorithms: Multi-omics integration is mostly limited to “data superposition” and lacks genuine systematic modeling; AI algorithms rely on large sample sizes and may be inapplicable to small sample cohorts [e.g., certain GCN models require thousands of samples (Ryan et al., 2025)]. Absence of Technical Standardization: There are significant variations in detection platforms and data analysis methods across different studies, which may lead to poor reproducibility of results. Many reported multi-omics analyses remain at the level of simple data juxtaposition, lacking genuine systematic joint modeling.

### 6.3 Translational challenges: model disconnect and insufficient clinical validation

#### 6.3.1 Disconnect between models and clinical practice

Commonly used cell models, animal models and other experimental models fail to fully recapitulate PD pathology. For instance, Xicoy et al. (2020) found that SH-SY5Y cells are not suitable for investigating the role of cholesterol in PD. Insufficient Clinical Validation of Biomarkers: Most biomarkers remain merely at the stage of cohort validation and have not progressed to clinical pilot application; for example, the 8-protein panel developed by Hällqvist et al. has not been tested in real clinical settings. In addition, some biomarkers are affected by pharmacological interventions [e.g., levodopa modulates the metabolome (Shao et al., 2021)], resulting in reduced clinical applicability. Slow Target Translation: Many potential therapeutic targets have been identified in basic research, yet very few have advanced to clinical trials. For example, targets such as GPNMB and ALOX5 have only completed *in vitro* and *in vivo* validation and not yet initiated human clinical trials. Furthermore, the underlying mechanisms of certain targets have not been fully elucidated—for example, how PARK7 mediates the clearance of glycolytic active intermediates (Heremans et al., 2022)—which imposes constraints on their translational potential.

## 7 Recommendations for future research directions

### 7.1 Biobank development: multicenter, multiethnic and longitudinal cohorts

(1) Construct a global multicenter PD omics database: integrate cohorts from Europe, Asia and Africa to cover populations with diverse genetic backgrounds and lifestyles, thereby enhancing the generalizability of research findings.
(2) Conduct large-scale longitudinal studies: conduct a follow-up duration of at least 10 years for patients in the preclinical stage and early-stage PD, collect multi-time-point biological samples (blood, CSF, fecal samples) and clinical data (motor and cognitive scores), and evaluate the long-term stability of biomarkers and their disease predictive value.
(3) Implement standardized sample processing: formulate unified sample collection, storage and preprocessing protocols to reduce heterogeneity, for example, develop a set of PD Omics Sample Processing Guidelines.

### 7.2 Technological innovation: high-sensitivity detection, AI integration and multimodal combination

#### 7.2.1 Single-omics technology upgrading

Develop ultra-high-sensitivity detection methods (e.g., nanopore mass spectrometry) to improve the detectability of low-abundance molecules; advance spatial omics technologies (e.g., spatial proteomics, spatial metabolomics) to elucidate the spatial distribution of pathological alterations in PD brain regions.

#### 7.2.2 Multi-omics integration algorithm optimization

Develop AI algorithms applicable to small sample sizes to enhance the efficiency of data integration; construct dynamic network models that integrate the gene-transcription-protein-metabolism-microbiota axis for the identification of core regulatory nodes.

#### 7.2.3 Multimodal combination

Omics data are combined with neuroimaging modalities (e.g., DaTscan, MRI) and clinical phenotypes to construct a multidimensional diagnostic model based on the ‘molecule-structure–function’ axis. For instance, on the basis of the MMT_CA model by Zhou et al. (2025), incorporate PET imaging data to improve the efficacy of early diagnosis.

### 7.3 Clinical translation: precision subtyping, target validation, and intervention exploration

#### 7.3.1 PD precision subtyping based on multi-omics

Integrate genetic factors (e.g., LRRK2, GBA mutations), molecular markers (e.g., pSer106 RAB12) and clinical phenotypes to establish a PD stratification system. For instance, classify PD into subtypes including LRRK2-associated, GBA-associated, sporadic motor-dominant, and sporadic cognitive-dominant subtypes, thereby providing a theoretical basis for personalized therapy.

#### 7.3.2 Clinical validation of therapeutic targets

Conduct prodromal assessments of safety and efficacy for therapeutic targets; promote the repurposing of approved drugs (e.g., ambroxol for PD-GBA patients) to shorten the translational cycle.

#### 7.3.3 Exploration of non-pharmacological interventions

Develop PD-specific probiotics and prebiotics based on the gut-brain axis mechanism; design combined exercise and dietary intervention regimens in light of lipid metabolism mechanisms and evaluate the efficacy of such interventions through multi-omics approaches.

## 8 Conclusion

From 2020–2025 (Figure 2), omics research on PD has centered on core objectives including elucidating pathogenic mechanisms, identifying clinical biomarkers, and discovering interventional targets. Driven by the technological evolution featuring the precision of single-omics, systematization of multi-omics, and standardization of auxiliary systems, this research has achieved the leap from scattered exploration to systematic integration. Multi-omics evidence provides in-depth support for the elucidation of pathogenic mechanisms, defining the interactive networks and stage specificity of core pathological mechanisms such as mitochondrial dysfunction, lipid metabolic dysregulation, *α*-Synucleinopathy, and gut-brain axis impairment. In the identification of clinical biomarkers, multi-omics has facilitated the upgrade from candidate molecules to clinical panels. The sample types have expanded from invasive CSF and brain tissue to minimally invasive plasma and urine, with a continuous improvement in analytical performance; meanwhile, the clinical utility of these biomarkers has extended from single diagnostic application to subtyping classification and therapeutic efficacy monitoring. In terms of interventional target discovery, multi-omics has identified multi-level potential targets involving signaling pathways, molecular entities, and gut microbes.

The period from 2020–2025 has been a pivotal six-year phase for PD omics research, witnessing its transition from scattered exploration to systematic integration. Research in this field has consistently centered on three core objectives: elucidating pathogenic mechanisms, screening biomarkers, and identifying potential therapeutic targets. Through the refinement of single-omics technologies, the systematization of multi-omics integration, and the standardization of supporting systems, the core molecular networks underlying PD pathogenesis have been gradually uncovered, a series of high-efficacy biomarkers have been screened out, and multiple potential intervention targets have been identified. A clear synergistic relationship has been established between technological evolution and the aforementioned research objectives: the enhanced sensitivity of single-omics technologies has facilitated the elucidation of pathogenic mechanisms from individual molecules to molecular pathways; the systematization of multi-omics integration has driven the advancement of biomarker screening from candidate molecules to clinical panels; and the standardization of supporting systems has boosted the translational progress of therapeutic targets from basic discovery to prodromal validation.

Despite the leap from scattered to systematic research achieved in PD omics, core limitations still restrict its clinical translational value. At the sample level, most studies still rely on small-sample, single-center cohorts, with a severe scarcity of data from diverse populations in Asia, Africa and other regions, and an insufficient number of long-term longitudinal follow-up studies. This results in inadequate validation of the generalizability and long-term effectiveness of research findings. At the technical level, single-omics technologies have obvious detection blind spots, such as the inability to accurately characterize structures like glycosidic bonds in lipids; multi-omics integration is mostly limited to data superposition, lacking genuine dynamic network modeling; in addition, the absence of standardization in detection platforms and data analysis methods remains a critical issue. At the translational level, cell and animal models fail to fully recapitulate the complex pathological features of PD; most biomarkers only progress to the stage of cohort validation and have not been tested in real-world clinical settings; potential therapeutic targets are still confined to the prodromal phase, leading to a prominent disconnect between basic research and clinical needs.

These limitations underscore the necessity for the field to guard against formalistic integration driven merely by technological advancement, and instead focus on sample representativeness, technical standardization and translational practicality, so as to effectively break down the barriers between laboratory research and clinical application. Moving forward, PD omics research must adhere to a clinical demand-oriented and technological innovation-supported approach. Through the construction of multicenter biobanks, the development of AI-based integration algorithms, and the exploration of precision subtyping and targeted interventions, it is imperative to facilitate the translation of research findings from the laboratory to the clinic. This will ultimately realize early diagnosis, precision therapy and disease modification for PD, thus bringing new hope to the PD patients worldwide. Furthermore, current AI-assisted multi-omics models predominantly focus on predictive performance metrics, while insufficient attention has been paid to overfitting risks caused by small cohort sizes and poor model explainability. Future studies need to adopt larger multi-ethnic longitudinal cohorts, conduct strict external validation, and employ interpretable machine learning algorithms to improve clinical applicability and biological interpretability.

## Data Availability

The original contributions presented in the study are included in the article/Supplementary material, further inquiries can be directed to the corresponding author.

## Supplementary material

SUPPLEMENTARY TABLE S2 Summary of representative multi-omics mechanistic studies.

SUPPLEMENTARY TABLE S3 Summary of representative biomarker panels and diagnostic performance.

SUPPLEMENTARY TABLE S4 Summary of candidate therapeutic targets from omics research.
