# Correction: Omics research in Parkinson's disease: evolution, integrated analysis, pathogenic mechanisms, biomarkers, and therapeutic targets

**DOI:** 10.3389/fnmol.2026.2012923

**Published:** 2026-10-13

**Authors:** Ruizhe Cao, Xue Bai, Huan Zhong

**Affiliations:** 1 School of Nursing, Tongji Medical College, Huazhong University of Science and Technology, Wuhan, China; 2 Department of Biochemistry and Molecular Biology, University of British Columbia, Vancouver, BC, Canada

**Keywords:** biomarkers, integrative multi-omics analysis, interventional targets, Parkinson's disease, pathogenic mechanisms

In the published article, the author Xue Bai was erroneously omitted as a corresponding author and was not included in the correspondence section as intended. The correct corresponding authors for this article are Xue Bai and Huan Zhong.

The original version of this article has been updated.

